# The Chromosome 18 Clinical Resource Center

**DOI:** 10.1002/mgg3.385

**Published:** 2018-03-30

**Authors:** Jannine D. Cody, Minire Hasi‐Zogaj, Patricia Heard, Annice Hill, David Rupert, Courtney Sebold, Bridgette Soileau, Daniel E. Hale

**Affiliations:** ^1^ Department of Pediatrics Chromosome 18 Clinical Research Center University of Texas Health Science Center at San Antonio San Antonio TX USA; ^2^ The Chromosome 18 Registry and Research Society San Antonio TX USA; ^3^ Department of Pediatrics Penn State Milton S. Hershey Medical Center Hershey PA USA

**Keywords:** 18p‐, 18q‐, chromosome 18, chromosome abnormalities, Ring 18, Tetrasomy 18p

## Abstract

**Background:**

The Chromosome 18 Clinical Research Center has created a pediatrician‐friendly virtual resource center for managing patients with chromosome 18 abnormalities. To date, children with rare chromosome abnormalities have been cared for either symptomatically or palliatively as a reaction to the presenting medical problems. As we enter an era of genomic‐informed medicine, we can provide children, even those with individually unique chromosome abnormalities, with proactive medical care and management based on the most contemporary data on their specific genomic change. It is problematic for practicing physicians to obtain and use the emerging data on specific genes because this information is derived from diverse sources (e.g., animal studies, case reports, in vitro explorations) and is often published in sources that are not easily accessible in the clinical setting.

**Methods:**

The Chromosome 18 Clinical Resource Center remedies this challenging problem by curating and synthesizing the data with clinical implications. The data are collected from our database of over 26 years of natural history and medical data from over 650 individuals with chromosome 18 abnormalities.

**Results:**

The resulting management guides and video presentations are a first edition of this collated data specifically oriented to guide clinicians toward the optimization of care for each child.

**Conclusion:**

The chromosome 18 data and guides also serve as models for an approach to the management of any individual with a rare chromosome abnormality of which there are over 1,300 born every year in the US alone.

## 1 INTRODUCTION

The birth prevalence of all chromosome abnormalities is one in 228 and those with unique chromosome imbalances account for about 17% of the total or one of every 1356 births (accounting for about 2900/year in the US) (Wellesley et al., 2012). However, these numbers are based on data acquired between 2000 and 2006. They likely underestimate the true frequency because contemporary technology is capable of detecting chromosome copy number changes several orders of magnitude smaller than was possible a decade ago. As a consequence there will be an ever greater proportion of children with complex medical needs who have a detectable and relevant chromosome change. Many of these chromosome changes will be rare and more will be individually unique. More importantly, once known, those changes will provide clues to help optimize clinical care. This is our goal as it specifically pertains to the chromosome 18 deletions and duplications and the rationale for developing rapidly accessible, dynamically collated treatment guides based on molecular‐level data.

Here, we present our recently created Chromosome 18 Clinical Resource Center. This virtual resource center includes medical management guides for the chromosome 18 abnormalities (18q‐, 18p‐, Ring 18, and Tetrasomy18p). In addition, to facilitate the use of these guides by clinicians we have created two free CME eligible presentations and two short YouTube tutorials. In total, these create a virtual resource center for providers about the chromosome 18 conditions (Table 1). This information is web‐based to optimize access to healthcare providers and to permit continuous updating as new information emerges (http://pediatrics.uthscsa.edu/centers/Chromosome18/chromosome18_clinical_management_guides.asp).

**Table 1:** The Chromosome 18 Clinical Research Center Resources

	Title	Format
18q Deletions	What is 18q‐? – 60 s summary	Webpage/PDF
	Distal 18q‐ Treatment and Surveillance	Webpage/PDF
	Proximal 18q‐ Treatment and Surveillance	Webpage/PDF
18p Deletions	What is 18p‐? 60 s summary	Webpage/PDF
	18p‐ Treatment and Surveillance	Webpage/PDF
Ring 18	What is Ring 18? 60 s summary	Webpage/PDF
	Ring 18 Treatment and Surveillance	Webpage/PDF
Tetrasomy 18p	What is Tetrasomy 18p? 60 s summary	Webpage/PDF
	Tetrasomy 18p Treatment and Surveillance	Webpage/PDF
CME Presentations	Caring for Patients with Chromosome 18 Abnormalities: An Overview	0.75 CME eligible presentation – 45 min
	Caring for Patients with Chromosome 18 Abnormalities: A Phenotype Walk	0.75 CME eligible presentation – 45 min.
“How to”	How to use the Chromosome 18 Gene Dosage Map	YouTube video – 12 min
	Gene Dosage Map Examples	YouTube video – 10 min
	How to create a personalized syndrome description	Webpage/PDF

The data used to create these Management Guides are from the participants at the Chromosome 18 Clinical Research Center and are based on in‐person assessments, medical and educational record review, and developmental survey data from 116 people with 18p‐, 343 people with 18q‐, 39 with Ring 18, and 70 with Tetrasomy 18p (Carter et al., 2015; Cody et al., 2015; Hasi‐Zogaj et al., 2015; O'Donnell et al., 2015). They reflect more than a quarter century of experience with affected individuals, many of whom we have followed since infancy. Our ultimate goal is to make the chromosome 18 abnormalities treatable conditions. As we move toward that goal, it is incumbent upon us to provide easily accessible and up‐to‐date guidance for busy clinicians and other healthcare providers who care for these children.

Management guides exist for more well‐known chromosome conditions such as Down syndrome and Williams syndrome. However, unlike Down syndrome or Williams syndrome, the chromosome 18 conditions are neither recurrent nor common. We believe that these guides can serve as models for other rare chromosome abnormalities as they address the three biggest challenges. First, chromosome abnormalities typically confer a complex and compound mix of medical and developmental issues that are seldom limited to a specific organ or metabolic pathway. Second, a large portion of individuals with chromosome abnormalities have very rare or even individually unique genomic copy number changes. Third, it is difficult for providers to stay abreast of the latest information, particularly when not all information about a particular condition applies to all patients with that condition. Despite those challenges, we have drawn on our quarter century of experience and research with the largest cohort ever reported—or likely to be reported—of individuals with chromosome 18 abnormalities. We hope to provide guidance regarding the medical management of individuals with these anomalies while establishing a dynamic format for incorporating new clinical and genetic information into their ongoing care.

**Figure 1:**
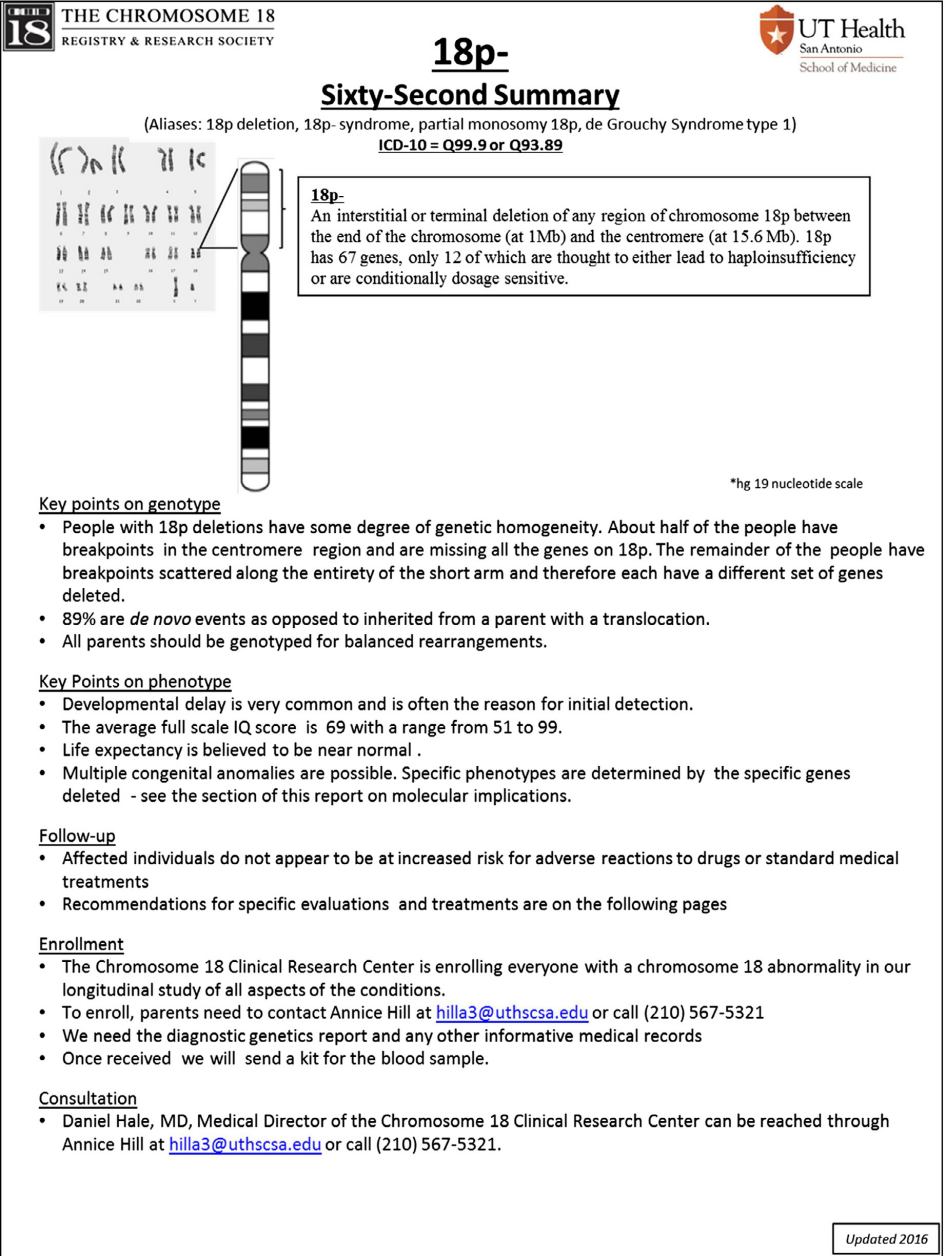
An example of the Sixty Second Summary page. This one is for 18p‐

The Management Guides are a novel approach to presenting directly relevant complex information that is useable ad libitum. We recognize that there are different levels of “need to know” and therefore designed three different levels, or tiers, of information. The most basic tier is introductory. This is a one‐page summary for each condition, or what we call the “Don't panic! it is only a chromosome” page. The brief overview is designed to introduce the provider to the chromosome 18 abnormality. The one‐page summary contains general information that will help inform interactions with the family and patient. For example, if the clinician needs to schedule a procedure on an affected teenager, they need to know whether the child is likely to be able to participate in their own care. An example of this page is shown in Figure 1.

The second tier or section is geared for use by a general provider, be it the neonatologist, the pediatrician, or general practitioner. The opening page is organized by the following sections:

- Potential conditions in a neonate
- Initial evaluations after diagnosis
- Initial referrals after diagnosis
- Closely monitor and manage
- Recommended annual screenings

**Figure 2:**
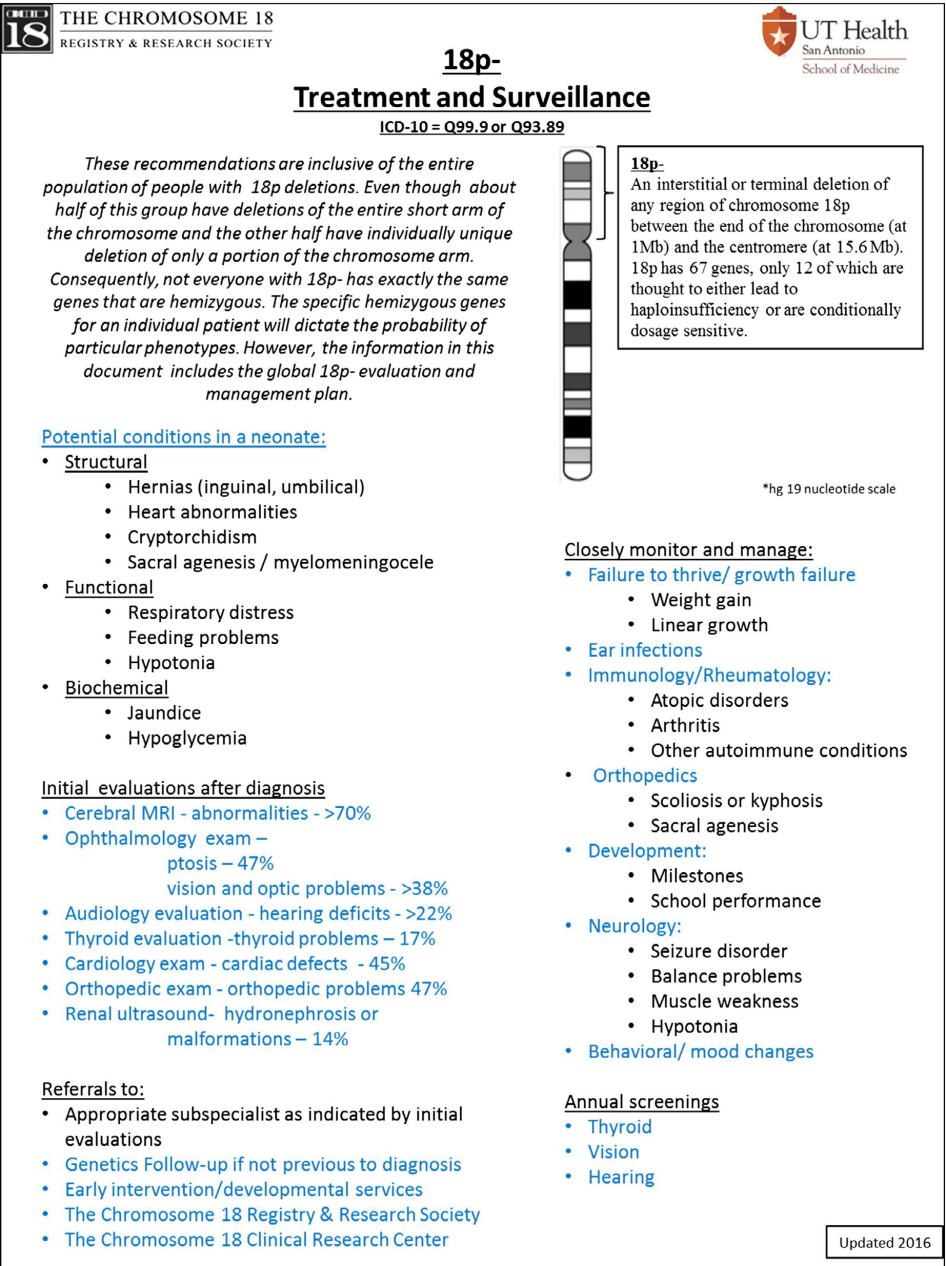
An example of the Treatment and Surveillance cover sheet. This example if for 18p‐. Each blue item is a hyperlink to additional information

A sample of this page for 18p‐ is shown in Figure 2. Each of the sections includes a list of potential features or medical issues. This page serves as the introduction to the entire document, a reminder of the need for ongoing monitoring, and a gateway to the system‐by‐system details. This page and the subsequent supporting pages can be printed as a PDF file. Hyperlinks are provided (in blue) that take the user to subsequent pages with more details which would be of interest to a specialist such as a cardiologist or endocrinologist.

A major challenge in organizing these treatment guides is that 18q‐, 18p‐, and Ring 18 are not classical syndromes. Classical syndromes have a single cause; however, every individual with 18q‐ (Heard et al., 2009) and Ring 18 (Carter et al., 2015) have different deletions and half of those with 18p‐ (Hasi‐Zogaj et al., 2015) have individually unique deletions. In fact, there are people with 18q deletions who share no common region of hemizygosity—one individual having a small terminal deletion and another individual having a proximal interstitial deletion. As a second example, approximately half of the population with 18p‐ have deletions that includes the entire short arm of chromosome 18, while the other half have unique (i.e., smaller) deletions. Thus, an individual with an 18p deletion who is missing only the distal portion of the short arm of 18p is not at increased risk for inflammatory bowel disease because the associated gene (*PTPN2*) (OMIM#176887) is located near the centromere. Despite the challenge of using the broad genetic delineation (e.g., 18q‐, 18p‐, Ring 18), we believe that a global overview is a useful tool which serves to narrow down the diagnostic differential.

The final section of the management guides distinguishes these guides from other traditional guides because it permits the creation of an individualized approach based on the molecular coordinates of a patient's deletion or duplication. This is possible due to unique Phenotype and Gene Dosage maps which we have created as custom tracks on the UCSC Genome Browser. Information on every gene on chromosome 18 is curated to determine its potential for being dosage sensitive. This process is updated on an ongoing basis and is completely reviewed annually. The 263 genes on chromosome 18 are currently designated as 194 dosage insensitive, 24 dosage sensitive, 24 conditionally dosage sensitive, and only 21 have too little information to make a determination. All the dosage classifications should be viewed as provisional because very few have sufficient human data to draw a definitive conclusion.

**Figure 3:**
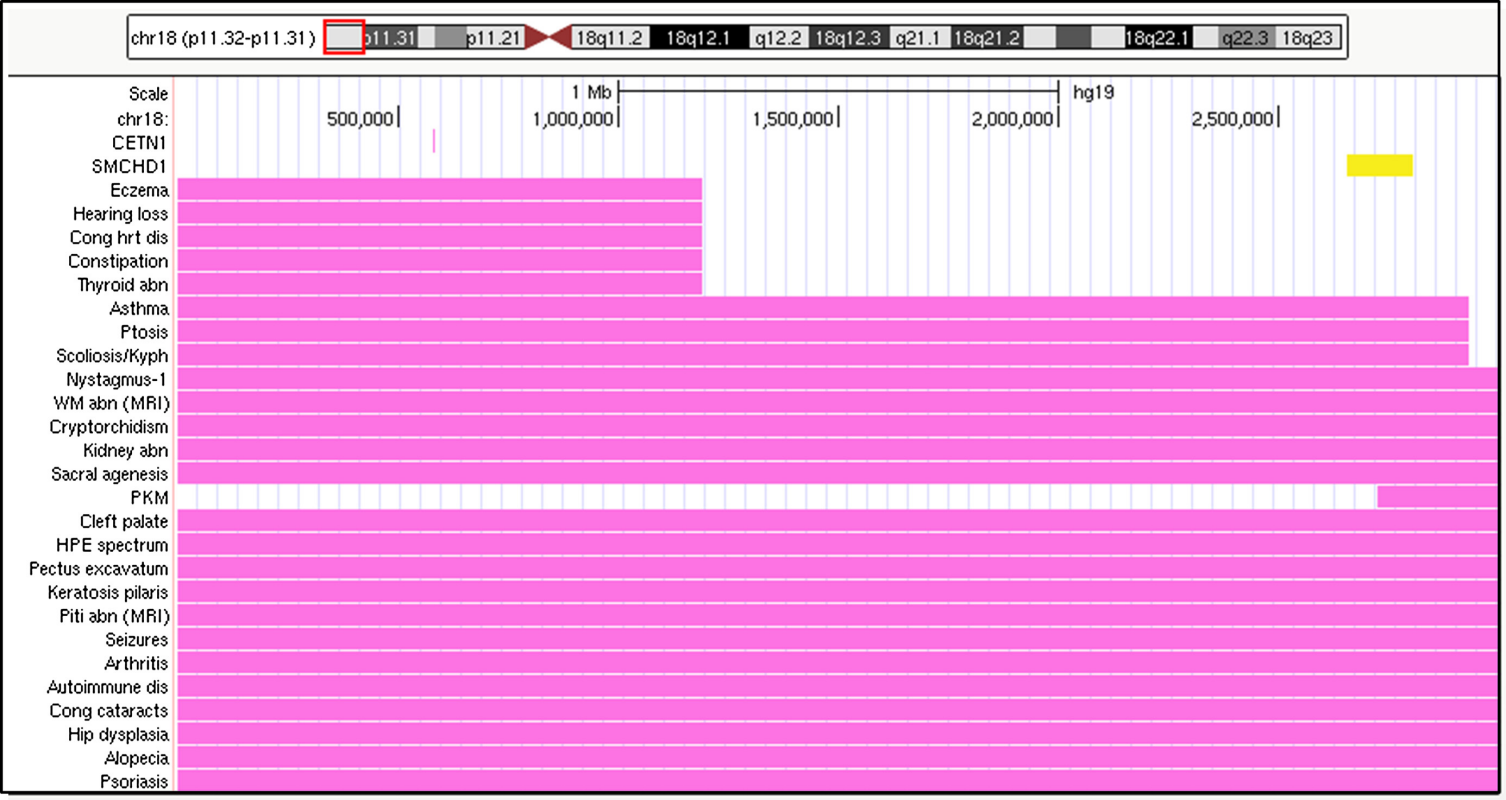
A partial screen shot of the Gene Dosage Map data for someone with a 3 Mb terminal deletion of 18p. Across the top is an ideogram of chromosome 18 with a (red) box indicating the portion of the chromosome shown beneath it. Below the ideogram, the top row indicates the scale of the image in megabases of DNA. The next row down shows the base pair positions within the image. The next two rows down indicate the positions of the two dosage‐sensitive genes in this region. By clicking on either of these the user is taken to a subsequent details page with additional information about why it is thought to be dosage sensitive and its effects. The next 26 rows indicate the phenotype regions for the phenotypes listed on the left side of the image. These phenotypes do not have an identified gene as the cause; however, the location of the causative gene has been narrowed to the region indicated by the (pink) bar

By following the directions in the section of the management guides labeled, “How to create a personalized syndrome description,” the exact base pair coordinates of the copy number change can be used to create a map view of the dosage sensitive genes and the phenotypes known to be associated with that region of the chromosome (Cody et al., 2009). In the case of 18p‐ used as an illustration here, the patient has a 3 Mb terminal deletion of 18p. Figure 3 shows that region of the chromosome on our gene dosage maps and is indicated by the red box around that section of the chromosome ideogram. In the bottom portion of the figure, the top row shows the scale of 1 Mb of DNA. The second row indicates the chromosome 18 nucleotide locations within the figure. The next two rows moving down the figure indicate that there are only two genes of the 14 in this region with potential clinical significance—*CETN1* (OMIM#603187) and *SMCHD1* (OMIM#614982). *CENT1* is shown in pink because it is classified as dosage sensitive and *SMCHD1* is shown in yellow because it is classified as conditionally dosage sensitive. Clicking on the gene will link to additional details pages with the latest data and commentary on the potential function of the gene and the consequences of a copy number change. Additionally, as shown in subsequent rows of data indicated by pink bars in the figure, there is a long list of associated phenotypes in this region for which the specific causative gene has not been identified. Again, in the website each of these genes or phenotypes can be clicked and are linked to the supporting data and literature. A short feedback and comment survey is also on the website so that future editions can be improved based on user feedback. (A note to the reader: because the website is periodically updated, the real‐time version may appear slightly different than the description here.)

The genomic diversity of people with chromosome 18 genomic copy number changes combined with the emerging data on the dosage sensitivity of individual genes provide a unique opportunity to demonstrate an approach to molecular‐based clinical utility. We think that these Management Guides provide both the general information as well as a guide to investigate the gene‐specific consequences of an individual patient's unique copy number change. Our goal is to provide physicians with a strategy to deliver the best most contemporaneous, care to children with chromosome 18 abnormalities.

These guides also support a specific diagnostic approach to chromosome 18 conditions. The emergence of clinical applications that are specific to the exact copy number variation provide support for the use of high‐resolution chromosome microarray analysis (CMA) as the most appropriate diagnostic tool. These tests are often denied by insurance companies because a molecular level determination of copy number changes provides no added clinical benefit above that of a less expensive microscopy‐based analysis. These management guides demonstrate the importance and clinical utility of CMA. As the science, and subsequently the medicine, progress and provide insight into the function and regulation of the specific dosage‐sensitive genes, it will become increasingly important for physicians to know exactly which genes their patient has in an abnormal copy number as this information will inform, if not dictate, treatment plans.

The gene‐specific information is particularly important for the families of children with chromosome 18 abnormalities. For most rare disorders, the diagnosis is an end point to the diagnostic odyssey; however, for many with chromosome abnormalities, the genomic diagnosis is just the beginning of this odyssey because the effects of the specific genes are largely unknown. What follows is usually a lifelong series of discoveries to determine all the consequences. Our goal here is to optimize the diagnostic odyssey and narrow the scope and depth so that families and their physicians are best prepared to facilitate a healthy and productive life for the affected individual.

Creating a management guide for a genetically heterogeneous chromosome condition is challenging. The number of potential chromosome imbalances is nearly infinite and requires novel ways of thinking about the practice of medicine. This requires leaving behind the notion of a unifying “syndrome” that relies on the description of clinical features only and presumes the same underlying genetic etiology. Rather, we must determine the effect of each individual gene involved in a deletion or a duplication and translate that into a personalized medical approach for a specific patient. We anticipate that some or even many features in a particular patient will be the result of a specific combination of dosage‐sensitive genes. Deciphering these relationships will be the task for many future studies.
